# Recognition and Relevance of Anti-DFS70 Autoantibodies in Routine Antinuclear Autoantibodies Testing at a Community Hospital

**DOI:** 10.3389/fmed.2018.00088

**Published:** 2018-04-09

**Authors:** John B. Carter, Sara Carter, Sandra Saschenbrecker, Bruce E. Goeckeritz

**Affiliations:** ^1^Lexington Medical Center, West Columbia, SC, United States; ^2^Institute for Experimental Immunology, Euroimmun AG, Luebeck, Germany

**Keywords:** antinuclear antibodies, autoantibody, autoimmunity, DFS70, LEDGFp75, systemic autoimmune rheumatic diseases

## Abstract

Antinuclear autoantibodies (ANA) displaying a dense fine speckled pattern (DFS, ICAP AC-2) on HEp-2 cells are frequently observed in clinical laboratory referrals, often associated with anti-DFS70 specificity. Anti-DFS70 positive patients rarely develop systemic autoimmune rheumatic disease (SARD), especially in the absence of clinical evidence or additional anti-extractable nuclear antigen (ENA) antibodies, prompting suggestions that an isolated DFS70-specific ENA may be an exclusionary finding for SARD. In this study, the frequency and diagnostic significance of anti-DFS70 autoantibodies was investigated in a community hospital cohort of patients undergoing routine ANA testing. ANA screening was performed by HEp-20-10-based indirect immunofluorescence, followed by ENA profiling using a multiparametric line immunoassay (LIA). Of 6,511 patient samples tested for ANA in 2016, the DFS pattern was identified in 1,758 (27.0%), 720 (41.0%) of which were anti-DFS70 positive by LIA. Of these, 526 (73.1%) revealed isolated anti-DFS70 reactivity, while 194 (26.9%) showed additional ENA specificities. Among 1,038 anti-DFS70 negative or borderline samples, 778 (75.0%) were ENA profile negative, while the remaining 260 (25.0%) showed a varied presence of other ENA specificities. Chart reviews of patients with an isolated anti-DFS70 ANA affirmed that ANA-related SARD is rare in the absence of clinical evidence or other ENA specificities, there being no case thus far identified. Rheumatoid arthritis patients occasionally had an isolated anti-DFS70 ANA and were positive for rheumatoid factor and anti-cyclic citrullinated peptide antibodies. In conclusion, the recognition of a DFS ANA pattern using a mitotic-rich HEp-2 substrate, followed by confirmation of anti-DFS70 specificity should be a routine ANA testing service. Use of an expanded ENA profile and clinical correlation is necessary to affirm the “isolation” of anti-DFS70 as the cause of an ANA. Recognition of isolated anti-DFS70 ANA enables reassurance of patients that SARD is unlikely, thus avoiding referral for more extensive testing. The presence of significant elevations of other ENAs may reflect SARD and warrants close clinical correlation and follow-up.

## Introduction

The presence of antinuclear autoantibodies (ANA) is one of the key diagnostic criteria of systemic autoimmune rheumatic diseases (SARD), such as systemic lupus erythematosus (SLE), Sjögren’s syndrome, systemic sclerosis, dermatomyositis/polymyositis (DM/PM), mixed connective tissue diseases, etc. (1, 2). Indirect immunofluorescence (IIF) using human epithelial (HEp-2) cells is the recommended “gold standard” for ANA screening as this substrate provides a variety of more than 100 native autoantigens including proteins, DNA, and ribonucleoproteins (2–4).

The dense fine speckled (DFS) nuclear pattern is one of the most common IIF patterns in the ANA screening routine of clinical diagnostic laboratories, often occurring in very high titers. The autoantibodies producing this pattern target the DFS protein of 70 kDa (DFS70), which is identical to the lens epithelium-derived growth factor or transcription co-activator p75 (LEDGFp75). DFS70/LEDGFp75 confers cell protection by regulating transcription of stress-related genes and is relevant to the pathophysiology of AIDS, cancer, autoimmunity, and inflammatory conditions. Anti-DFS70 autoantibodies might play protective, pathogenic, or sensor roles (5–13). The International Consensus on ANA Patterns (ICAP) committee has recently classified the DFS pattern as “AC-2” competency level recognition pattern, defined by a dense and heterogeneous speckled staining in the nucleoplasm of interphase cells (sparing the nucleoli) and the metaphase chromosomal plate (14, 15). Recognition of this pattern on HEp-2 substrates is challenging as it can be confused with other nuclear patterns or may occur in the context of another clinically relevant ANA, and because IIF interpretation is dependent on technician expertise (16–19). Thus, a positive DFS IIF result has to be followed by a monospecific immunoassay (e.g., ELISA, CLIA, immunoblot, immunoadsorption) (20) to accurately confirm the presence of anti-DFS70 autoantibodies, as recommended in diagnostic algorithms (19, 21–26).

The clinical significance of anti-DFS70 autoantibodies is not clear due to the absence of disease specificity (9, 19, 22, 26–29). Regardless of the detection method, DFS ANA and/or anti-DFS70 antibodies have been detected at elevated frequency in apparently healthy individuals (0–21.6%) (13, 16, 30–43), but also in routine ANA screening cohorts (0.3–16.6%) (21, 22, 34, 36, 40, 41, 43–49), and various non-SARD inflammatory and neoplastic conditions (3.3–71.4%; e.g., Vogt–Harada syndrome, atopic dermatitis, psioriasis, interstitial cystitis, Hashimoto’s thyroiditis, ocular diseases, chronic fatigue syndrome asthma, or prostate cancer) (13, 30–32, 34, 37, 40, 50–55). In contrast, they are rare in patients with SARD (0–28.6%) (13, 16, 33–35, 43, 45, 51, 52, 55–57), showing an overall frequency of only 2.8–4.5%, which is remarkably lower than in healthy individuals and control cohorts (19, 28). Anti-DFS70 reactivity in SARD is usually accompanied by additional SARD-related antibodies, while isolated anti-DFS70 reactivity in SARD reportedly amounts to only 0.5–0.7% (19, 28). Thus, antibodies to DFS70 are increasingly regarded as a negative predictive biomarker for excluding the diagnosis of SARD, particularly in the absence of clinically relevant ANA (16, 19, 23, 28, 33, 34, 53, 58–61). This is supported by studies reporting on healthy individuals with isolated anti-DFS70 reactivity who did not develop SARD within a follow-up of 3–4 years (“benign autoimmunity”), and by a likelihood ratio (LR+) for the absence of SARD of 10.9 ascribed to isolated reactivity to DFS70 (16, 42, 59).

A few recent surveys have examined the prevalence of DFS ANA in sera submitted for routine ANA screening, but only some of these used anti-DFS70 assays to confirm the antibodies’ specificity in all or in just a subset of tested samples. Considering also the diverse composition of the screened cohorts as well as the differences in assays and IIF interpretation, the currently available data have to be interpreted with caution (19, 28). Thus, the objective of this retrospective study was to investigate the frequency of anti-DFS70 autoantibodies among consecutive sera referred for ANA testing in a community hospital laboratory, using HEp-20-10 IIF for antibody screening followed by a multiparametric line immunoassay (LIA) to confirm or disprove the presence of anti-DFS70 and/or other autoantibodies in all samples displaying a DFS ANA pattern. Chart review of medical records and clinical follow-up allowed for evaluation of associated clinical associations and diagnostic relevance.

## Materials and Methods

### Patients and Serum Samples

We studied 6,511 serum samples from patients presenting at the Lexington Medical Center, a 400 bed acute-care community hospital with a strong rheumatology service, in West Columbia, SC, USA. These samples were collected for routine ANA testing, within a 1-year period. Individual and ethical approval was not mandatory for this study as patient data and samples were used anonymously to maintain confidentiality. Serological analyses were performed blinded to clinical data.

### IIF Assay

ANA screening was performed using the IFA40: HEp-20-10 kit assay (Euroimmun, Luebeck, Germany). Testing and evaluation were carried out according to the manufacturer’s instructions. In brief, microscope slides containing millimeter-sized biochips coated with HEp-20-10 cells were incubated with serial serum dilutions (starting with 1:40 in PBS-Tween) for 30 min at room temperature, washed with a flush of PBS-Tween, and immersed in PBS-Tween for 5 min. For detection of bound antibodies, fluorescein isothiocyanate-conjugated goat anti-human IgG was applied for 30 min at room temperature, followed by washing as described before. After embedding in mounting medium, the slides were cover-slipped and evaluated by two independent observers using fluorescence microscopy. Sera displaying ANA fluorescence patterns at a titer ≥1:40 were considered positive. All DFS ANA pattern results were reported as possibly DFS70-related, with extractable nuclear antigen (ENA) follow-up analysis recommended.

### Immunoblot Assay

Specific profiling for anti-ENA autoantibodies was performed using the EUROLINE ANA profile 3 plus DFS70 (IgG) kit according to the manufacturer’s instructions (Euroimmun). The blot strips contain 16 separate antigens, 11 of which are native purified proteins (nRNP/Sm, Sm, SS-A, SS-B, Scl-70, Jo-1, dsDNA, nucleosomes, histones, ribosomal P-protein, and AMA M2) and 5 of which are recombinant antigens (Ro-52, PM-Scl, CENP B, PCNA, and DFS70). The DFS70 antigen is a full-length recombinant protein (amino acids 1–530) expressed in mammalian cells. The EUROBlotOne system (Euroimmun) was used for automated processing of all incubation and washing steps. Buffer-soaked blot strips were incubated with 1:101 diluted serum for 30 min at room temperature. After three washing cycles of 5 min each, binding of specific antibodies was detected by incubation with alkaline phosphatase-conjugated goat anti-human IgG for 30 min at room temperature. Subsequently, the strips were washed and then incubated with nitrobluetetrazoliumchloride/5-bromo-4-chloro-3-indolylphosphate (NBT/BCIP) for 10 min, followed by the addition of distilled water to stop the reaction. The incubated strips were automatically dried and photographed. Band intensities were evaluated by using the EUROLineScan software (Euroimmun). Signal intensities ≥15 were considered positive, 8–14 borderline, and ≤7 negative.

## Results

### IIF Screening and ANA Differentiation

**Figure 1:**
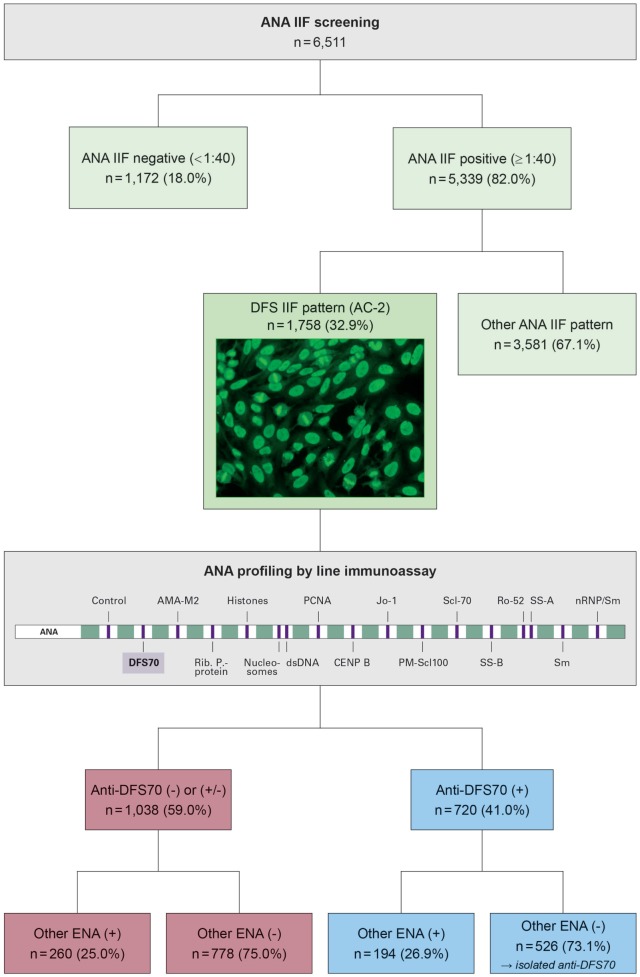
Results of antinuclear autoantibody (ANA) testing in 6,511 patients samples, focusing on a subgroup of 1,758 samples with a dense fine speckled (DFS) pattern (AC-2) in indirect immunofluorescence (IIF). The DFS pattern is characterized by staining of dense fine speckles in interphase nuclei and strongly fluorescent mitotic chromosomes. Initial ANA screening was performed using IIF on HEp-20-10 cells. Samples showing a DFS IIF pattern were analyzed for specific autoantibody reactivity using a line immunoassay (LIA) containing 16 relevant antigens. Frequencies (percentage values in brackets) refer to the number of patients in the respective next higher subgroup. (+), positive; (±), borderline; (−), negative.

**Figure 2:**
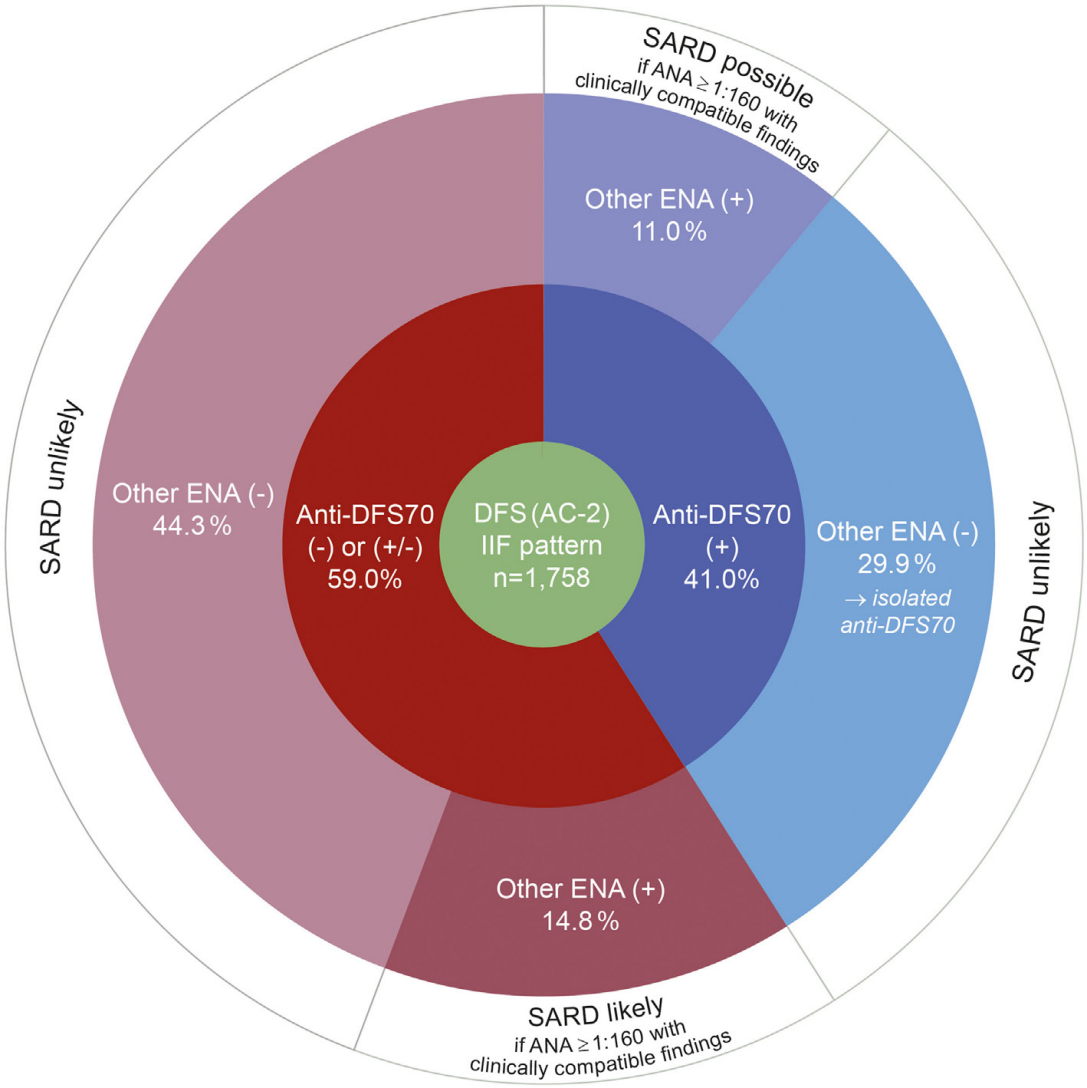
Results of autoantibody profiling by line immunoassay in serum samples with a typical dense fine speckled (DFS) pattern (AC-2) in indirect immunofluorescence (IIF). Percentages for anti-DFS70 positive (blue) and negative (red) results by LIA refer to the total number DFS IIF positive cases (green). The likelihood for the presence or development of an associated systemic autoimmune rheumatic disease (SARD), in accordance with Conrad et al. (19), is indicated in the outermost circle (white). (+), positive; (±), borderline; (−), negative.

Among 6,511 serum samples tested for ANA in 2016, 5,339 (82.0%) were ANA positive by IIF at a titer ≥1:40 and 1,172 (18.0%) were negative (<1:40). A DFS, AC-2 IIF pattern was identified in 1,758 sera, corresponding to 27.0% of all samples tested, and 32.9% of ANA positive samples. Analysis of the DFS positive samples by LIA revealed the presence of anti-DFS70 autoantibodies in 720 (41.0%) of all DFS, AC-2 pattern ANAs. Among these, 526 (73.1%) had isolated anti-DFS70 reactivity, whereas the remaining 194 (26.9%) showed additional ENA specificities. 1,038 (59.0%) of the samples with a DFS IIF pattern were found anti-DFS70 negative (or borderline) by LIA, including 260 (25.0%) samples with a varied presence of other ENA specificities and 778 (75.0%) ENA profile negative samples (Figure 1). Thus, isolated anti-DFS70 reactivity was detectable in 29.9% of all DFS IIF positive samples, suggesting a low probability for the presence or development of SARD (Figure 2).

### Diagnostic Relevance of Anti-DFS70 Positivity

Rheumatologist chart reviews of anti-DFS70 positive patients were performed to examine if the finding of isolated anti-DFS70 reactivity is useful as a rule-out for SARD. Thus far, no case of ANA-related SARD has been identified at our hospital among patients positive for isolated DFS70-specific autoantibodies. In contrast, the additional presence of other ENAs at a significant level has clinical relevance specific to those autoantibodies, as reflected by representative cases given in Table 1. Occasionally, we found rheumatoid arthritis patients with isolated anti-DFS70 ANA to be positive for rheumatoid factor and antibodies against cyclic citrullinated peptide (anti-CCP).

**Table 1:** Characteristics of anti-DFS70 positive patients (aged between 26 and 69 years) with additional high-level autoantibodies (representative cases).

Patient ID	Clinical diagnosis	Autoantibodies against (specific results)
1	Uveitis	DFS70 (166), PM-Scl100 (86)
2	Systemic lupus erythematosus (SLE)	DFS70 (72), Histones (24), Chromatin (42), RNP (70), SS-A (74)
3	Raynaud’s disease, polyarthralgias	DFS70 (118), Scl-70 (103)
4	SLE	DFS70 (170), DNA (45), CENP B (48)
5	Discoid lupus erythematosus, rheumatoid arthritis	DFS70 (202), CENP B (70)
6	Lupus, olfactory, visual and auditory hallucinations	DFS70 (170), Ro-52 (95), SS-A (84)
7	Sicca syndrome, Raynaud’s disease, myalgia, and myositis	DFS70 (156), PM-Scl100 (27), RNP (18)
8	SLE with other organ involvement	DFS70 (110), Ro-52 (155), SS-A (110)
9	SLE	DFS70 (176), SS-B (22), Sm (23)
10	Sjögren’s syndrome	DFS70 (169), SS-A (50)

*Anti-DFS70, autoantibodies against the dense fine speckled protein of 70 kDa (identical to LEDGFp75, lens epithelium-derived growth factor or transcription co-activator p75)*.

## Discussion

For many years, we noted a high number of samples containing often high-titer finely speckled ANAs but with a negative ENA profile. When a mitotic-rich HEp-2 cell substrate (HEp-20-10) became available, we realized that the majority of these finely speckled ANAs also showed a positive mitotic fluorescence, i.e., a mixed speckled/homogeneous pattern, later termed as “dense fine speckled,” which was often associated with anti-DFS70 specificity. Despite the fact that the DFS IIF pattern has recently been classified for competent-level reporting by ICAP (AC-2 pattern) due to its negative association with SARD (14), it is still not widely recognized among commercial laboratories, and often reported as “speckled,” “homogeneous,” or even “negative,” with no follow-up testing available. However, confirmatory tests are necessary in light of sustained concern that the recognition of the DFS pattern is challenging and prone to mistakes (18, 21, 22, 24, 25, 62). In this study, we examined the prevalence of anti-DFS70 autoantibodies and their association with ANA-related SARDs in a community hospital routine ANA referral cohort by using an expanded ENA profile for follow-up testing of samples with a DFS IIF pattern.

We observed the DFS IIF pattern in 27.0% of all samples submitted for ANA screening. Other groups reported the presence of this pattern in 0.3–16.6% of non-selected routine ANA cohorts, resulting in an overall frequency of about 7.7%, as indicated in Table 2 (19, 21, 34, 36, 40, 41, 43–47, 49). Reasons for the wide range of positivity rates are speculative but may include heterogeneity in the composition of the studied ANA screening cohorts (e.g., gender, age, ethnicity, reasons for requesting ANA tests) as well as differences in the screening method and in defining an appropriate cut-off value (26). Of note, the analytical performance of HEp-2 IIF crucially depends on the quality of the substrate and assay, particularly with regard to the ratio of mitotic versus interphase cells, cell morphology, reproducibility, and dilution linearity. The HEp-20-10 substrate (a variant of the standard HEp-2 cell line with a 10-fold higher number of mitotic cells), as used in the present study, has been reported to facilitate the recognition, discrimination and titer estimation of ANA patterns (63).

**Table 2:** Frequency of DFS IIF (ICAP AC-2) and/or anti-DFS70 antibodies in unselected routine ANA screening cohorts, as determined in different studies.

Reference	ANA IIF	DFS IIF pattern		Anti-DFS70 reactivity			
	Positive/total	Positive/total	Positive/ANA positive	Positive/total	Positive/ANA positive	Positive/DFS IIF positive	Detection method
Dellavance (44)	13,641/30,728 (44.4%)	5,089/30,728 (16.6%)	5,089/13,641 (37.3%)	ND	ND	80/81 (98.8%)	WB
Bizzaro et al. (40)	ND	172/21,516 (0.8%)	ND	ND	ND	ND	–
Kang and Lee (45)	352/2,654 (13.3%)	101/2,654 (3.8%)	101/352 (28.7%)	ND	ND	ND	–
Pazini et al. (46)	790/2,788 (28.3%)	29/2,788 (1.0%)	29/790 (3.7%)	ND	ND	ND	–
Mahler et al. (34)	ND	53/3,263 (1.6%)	ND	53/3,263 (1.6%)	ND	53/53 (100%)	CLIA/ELISA
Bizzaro et al. (22)	ND	ND	ND	2/155 (1.3%)	ND	ND	CLIA
Marlet et al. (41)	ND	421/16,754 (2.5%)	ND	ND	ND	ND	–
Schmeling et al. (36)	55/200 (27.5%)^a^	ND	ND	9/200 (4.5%)^a^	9/55 (16.4%)^a^	ND	CLIA
Sener and Afsar (49)	1,302/5,800 (22.4%)	16/5,800 (0.3%)	16/1,302 (1.2%)	ND	ND	ND	–
Lee et al. (47)	ND	181/10,528 (1.7%)	ND	109/10,528 (1.0%)	ND	109/181 (60.2%)	ELISA
Mutlu et al. (21)	368/1,786 (20.6%)	90/1,786 (5.0%)	90/368 (24.5%)	ND	ND	62/74 (83.8%)	ELISA
Shovman et al. (43)	ND	ND	ND	13/85 (15.3%)	ND	ND	CLIA
Carter et al. (present study)	5,339/6,511 (82.0%)	1,758/6,511 (27.0%)	1,758/5,339 (32.9%)	720/6,511 (11.1%)	720/5,339 (13.5%)	720/1,758 (41.0%)	LIA

Total	21,847/50,467	7,910/102,328	7,083/21,792	906/20,742	729/5,394	1,024/2,147	
Overall frequency (95% CI)	43.3% (42.9–43.7%)	7.7% (7.6–7.9%)	32.5% (31.9–33.2%)	4.4% (4.1–4.7%)	13.5% (12.6–14.5%)	47.7% (45.6–49.8%)	
Range frequency (%)	13.3–82.0%	0.3–27.0%	1.2–37.3%	1.0–15.3%	13.5–16.4%	41.0–100%	

*ANA, antinuclear autoantibodies; Anti-DFS70, autoantibodies against the dense fine speckled protein of 70 kDa (identical to LEDGFp75, lens epithelium-derived growth factor or transcription co-activator p75); CLIA, chemiluminescence immunoassay; DFS, dense fine speckled; ELISA, enzyme-linked immunosorbent assay; ICAP, International Consensus on ANA Patterns; IIF, indirect immunofluorescence; LIA, line immunoassay; ND, not determined; WB, Westernblot*.

*^a^Pediatric cohort (≤18 years)*.

Apart from the difficulties in recognizing the DFS IIF pattern, the high prevalence of anti-DFS70 autoantibodies in healthy individuals, lack of association with a particular disease group, and negative association with SARD necessitate the confirmation of anti-DFS70 reactivity (26). In the present study, 41.0% of samples with a DFS IIF pattern were confirmed to contain anti-DFS70 autoantibodies using the EUROLINE ANA Profile 3 plus DFS70. Bizzaro et al. reported for this LIA a sensitivity of 51.6% (specificity 96.6%) with respect to a presumptive DFS IIF pattern on HEp-2 cells, which was similar to other assays based on DFS70 truncated to the C-terminal major epitope region (CLIA, 43.5%; dot blot, 51.6%) (24, 64). In other surveys, the concordance rates between DFS IIF and specific anti-DFS70 assays (e.g., CLIA, ELISA, Westernblot) were highly divergent, ranging between 14% (17) and >90% (34, 44, 48). Our data are consistent with previous findings in that a subset of sera with a typical DFS IIF pattern does not show anti-DFS70 reactivity using a more specific method. Vice versa, samples with anti-DFS70 positivity by a monospecific assay may not clearly show a DFS IIF pattern (17, 56). Factors potentially contributing to these inconsistencies include: (1) Heterogeneity among the anti-DFS70 autoantibodies that cause a typical DFS pattern on HEp-2 cells but not all of which react with the antigenic substrate applied in the confirmatory assays. (2) Reactivity of the recombinant antigen is slightly affected by the procedure of antigen preparation, coating (loss of conformational epitope), and choice of the cut-off value. (3) A DFS-like ANA IIF pattern may be produced by antibodies directed against nuclear antigen(s) other than DFS70, particularly interacting partners of this autoantigen or proteins sharing the same cellular localization, such as methyl CpG-binding protein 2 (MeCP2), pogo transposable element-derived protein with zinc finger (PogZ), c-Myc-interacting protein JPO2, or Cdc7-activator of S-phase kinase (Cdc7-ASK) (65–68). For example, MeCP2 was found to co-localize with DFS70 in the nucleus and to produce a DFS-like HEp-2 staining pattern, which is not affected by pre-adsorption of anti-DFS70 antibodies (24, 62).

According to a recent meta-analysis, the mean prevalence of isolated anti-DFS70 autoantibodies is only about 0.7% in SARD patients and that of anti-DFS70 accompanied by SARD-specific ENA is 3.8% (28). In contrast to this, isolated anti-DFS70 positivity is associated with a likelihood ratio (LR+) of 10.9 for the absence of SARD (59). Referring to the diagnostic algorithm recommended by Conrad et al. (19), 74.2% of our patients with a DFS IIF pattern can be classified as unlikely for the presence or development of SARD, either by the presence of isolated anti-DFS70 reactivity or by the absence of SARD-associated and DFS70-specific ENA (Figure 2). Gundin et al. reported that none of their patients with an isolated positive anti-DFS70 result developed SARD during a 10-year follow-up (60). Based on chart reviews, we also found no clinical evidence of active SARD in cases with isolated anti-DFS70 positivity. Rather, the additional presence of other ENA specificities at high levels has clinical relevance specific to those autoantibodies. While borderline elevations are not likely relevant for diagnostics, the presence of additional high-titer ENAs warrants close clinical correlation and follow-up. This observation conforms with the current classification of anti-DFS70 autoantibodies as a potential negative predictive biomarker of SARD in the absence of other clinically relevant ENA (16, 19, 23, 28, 33, 34, 53, 58–61). However, the presence of anti-DFS70 antibodies does not replace diligent ENA differentiation. If an ANA pattern is observed using IIF, elaborate monospecific ENA differentiation needs to be conducted in any case without exceptions, independently of whether antibodies against DFS70 are present or not. Only when no disease-relevant ENAs are detected after the specialized diagnostics, a positive DFS70 result can help to explain the observed IIF pattern. If confirmatory testing by means of a multiparametric assay (“multiplexing”) (20) did not reveal a relevant antibody, the presence of other ENA cannot be ruled out and should be considered especially in patients with sustained suspicion of SARD. As discussed in a recent study (60), the number of follow-ups may be reduced to one or two per year in patients with a positive ANA IIF result but negative assays for both ENA and anti-DFS70. Through a reduction in the number of follow-up antibody testing and outpatient clinic visits, this contributes to substantially increased cost-efficiency (60), which conforms to our experience.

Due to increasing workload, some laboratories have switched their ANA screening to largely automated multiplex methods (69), with the option of using IIF as a second step. Because of the limited number of purified/recombinant antigens, multiplex assays focus on clinically significant ANAs, but also lack sensitivity compared to HEp-2 cells, leading to an estimated 35% of SARD patients with false negative screening results with respect to IIF (4). Accordingly, the American College of Rheumatology (ACR) and international committees recommend HEp-2 IIF as the standard screening method for ANA detection (2–4), reinforcing the outstanding potential of this technique. In cases of strong clinical suspicion and negative alternative methods, it is mandatory to perform IIF. This recommendation is followed in the present and earlier studies that include anti-DFS70 testing.

The diagnostic algorithm presented here conforms to several previously reported algorithms incorporating anti-DFS70 in that any sample revealing a (suspected) DFS ANA pattern in HEp-2 IIF screening should undergo confirmatory tests for anti-DFS70 and SARD-associated autoantibodies (Figure 2) (23, 26, 28, 60). Results are to be reported to the clinician with a comment on the diagnostic relevance of the serological result, pointing also on the significance of clinical findings and other laboratory tests.

In conclusion, DFS70 ANAs are common in a community hospital patient population. Recognition of the DFS IIF pattern using a mitotic-rich HEp-2 cell substrate, followed by confirmation of DFS70 specificity should be a routine ANA testing service. Use of an expanded ANA/ENA panel that includes a specific anti-DFS70 test, and close clinical correlation, is necessary to confirm the “isolation” of anti-DFS70 as the cause of an elevated ANA titer. Significant elevations of other ENAs in addition to anti-DFS70 may reflect SARD, and warrant close clinical correlation and follow-up testing. This testing enables reassurance of DFS70 ANA-positive patients who may otherwise be referred for extensive testing for autoimmune disease. An isolated DFS70 ANA does not “exclude” SARD, as that is largely a clinical diagnosis, supported by laboratory evidence. It is simply not a diagnostic point supportive of a SARD diagnosis. As many clinicians are not yet familiar with DFS70 ANAs, all related findings should be explained clearly in laboratory reports.

## Ethics Statement

Individual and ethical approval was not mandatory for this study as patient data and samples were used anonymously to maintain confidentiality.

## Author Contributions

SC and JC designed and managed the study. BG was responsible for rheumatologic diagnostic assessment. SC and JC performed the laboratory analyses, with results interpretations and collated all data. JC provided medical staff consultation and advice regarding DFS70 ANA test results. JC, SC, SS and BG contributed to writing of the manuscript and approved the final version.

## Conflict of Interest Statement

SS is an employee of Euroimmun AG, a company that develops and produces immunoassays for the detection of disease-associated antibodies. The other authors have nothing to disclose.
